# Efficacy of Weekly Split versus Single Doses of Ergocalciferol on Serum 25-Hydroxyvitamin D among Patients on Continuous Ambulatory Peritoneal Dialysis: A Randomized Controlled Trial

**DOI:** 10.1155/2021/5521689

**Published:** 2021-03-13

**Authors:** Naowanit Nata, Jessada Kanchanasinitth, Pamila Tasanavipas, Ouppatham Supasyndh, Bancha Satirapoj

**Affiliations:** Division of Nephrology, Department of Medicine, Phramongkutklao Hospital and College of Medicine, Bangkok, Thailand

## Abstract

**Background:**

Vitamin D deficiency is a common problem among patients on continuous ambulatory peritoneal dialysis (CAPD). Vitamin D supplementation leads to reduced serum parathyroid hormone levels and improved cardiovascular markers. Different doses and time intervals of oral vitamin D supplementation may differ in each patient on dialysis. The study aimed to evaluate the efficacy of weekly split and single dose of ergocalciferol at 60,000 IU on serum 25-hydroxyvitamin D (25(OH)D) among patients on CAPD.

**Methods:**

A randomized study was conducted among patients on CAPD with vitamin D deficiency or insufficiency (25(OH)D < 30 ng/mL). Patients were randomly assigned to two groups: the split dose group was given ergocalciferol 20,000 IU three times weekly and the single dose group was given ergocalciferol 60,000 IU once weekly for 8 weeks. Main outcomes measured serum 25(OH)D concentrations, serum calcium, serum phosphate, and intact parathyroid levels at 8 weeks after being enrolled.

**Results:**

Of 128 screened patients, 50 met the criteria for eligibility and were randomized. At 8 weeks after treatment, mean serum 25(OH)D concentrations significantly increased from baseline 22.7 ± 5.9 to 29.5 ± 9.5 ng/mL (*P*=0.004) in the split dose group and 22.9 ± 5.3 to 31.2 ± 12.3 ng/mL (*P*=0.003) in the single dose group. No significant change was found in increase of serum 25(OH)D between the two groups (*P*=0.561). At the end of study, a similar proportion of patients in both groups reached the desirable serum concentration of 25(OH)D ≥ 30 ng/mL (60% in the single group vs. 40% in the split group, *P*=0.258). No significant cases of hypercalcemia, hyperphosphatemia, or serious adverse events occurred during the study.

**Conclusion:**

Weekly single and split doses of ergocalciferol 60,000 IU achieved similar effects on serum 25(OH)D levels among patients on CAPD with vitamin D insufficiency or deficiency, suggesting that weekly single dose would be prescribed for adequate vitamin D repletion. This trial is registered with TCTR20200821005.

## 1. Background

Among patients with advanced chronic kidney disease (CKD) and end-stage renal disease on dialysis, the incidence and prevalence of vitamin D deficiency and insufficiency has been reported to be higher than that of the general population [1–3]. The clinical impact of low serum 25-hydroxyvitamin D (25(OH)D) level among patients with CKD has been associated with secondary hyperparathyroidism, decreased bone mineral density, increased muscle weakness and risk of falls, increased risk for mortality, and progression to dialysis [4, 5]. Vitamin D supplementation among patients with CKD and on dialysis leads to decreased serum parathyroid hormone (PTH) level, reduced proteinuria, improved endothelial cardiovascular markers, and decreased inflammatory markers [6, 7]. Current systematic review and meta-analysis in CKD patients indicated that vitamin D treatment was associated with reductions in all-cause mortality in the observational studies, but significant association was not found in the randomized controlled studies [8]. Ongoing debate embraces which dose and type of vitamin D supplement should be used for supplementation, ergocalciferol or cholecalciferol, depending on availability [9].

National Kidney Foundation-Kidney Disease Outcomes Quality Initiative (NKF-KDOQI) guidelines currently recommend vitamin D supplementation in advanced CKD patients with 25(OH)D levels <30 ng/mL [10]. Standard dose of ergocalciferol as recommended by K/DOQI guidelines was inadequate for correcting vitamin D deficiency and insufficiency in CKD patients [11], whereas high dose ergocalciferol was safe and significantly increased serum 25(OH)D level in advanced CKD patients [12]. Our study started initial high dose of ergocalciferol at 60,000 IU/week independent on baseline serum 25(OH)D level in all CAPD patients with vitamin D deficiency and insufficiency. Ergocalciferol is widely available in Thailand for oral use in 20,000 IU capsules. No strong evidence is available about drug administration, but ergocalciferol (20000 IU) was prescribed orally three times weekly or once a week with total dose of 60,000 IU/week in general practice [13]. However, to date, limited clinical trials and published literature have compared the efficacy of single or split high dose of ergocalciferol among patients on continuous ambulatory peritoneal dialysis (CAPD). This study aimed to examine the efficacy of single and split dose ergocalciferol weekly to treat vitamin D deficiency and insufficiency among patients on CAPD.

## 2. Methods

### 2.1. Study Design

**Figure 1:**
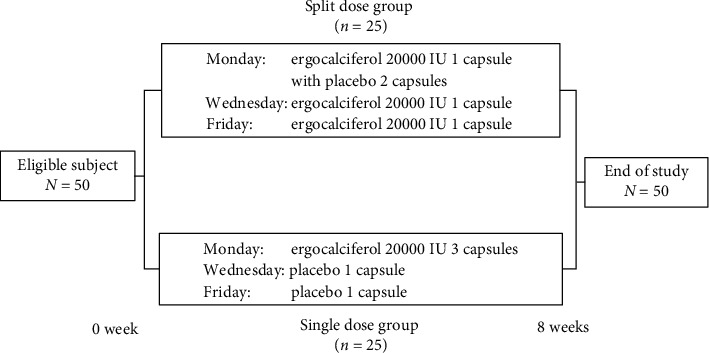
Study design.

This single-center, prospective, double-blind, randomized controlled study was conducted in Phramongkutklao Hospital from January 2018 to February 2019. The study was approved by the Ethics Committee of the Institute Review Board of the Royal Thai Army Medical Department, and all subjects provided written informed consent. The study complies with the Declaration of Helsinki. The study was registered at Thai Clinical Trials Registry (TCTR) (TCTR20200821005). Enrolled participants were randomly assigned to two groups at a ratio of 1 : 1; the split dose group was given ergocalciferol 20,000 IU three times weekly and the single dose group was given ergocalciferol 60,000 IU weekly for eight weeks, as illustrated in Figure 1.

### 2.2. Study Population

The inclusion criteria comprised patients on CAPD for at least three months before enrolling, aged ≥18 years, and having received a diagnosis of vitamin D deficiency defined by serum 25(OH)D less than 20 ng/mL or vitamin D insufficiency defined by 20 to 29 ng/mL. Subjects with a history of liver diseases, hypocalcemia, hypercalcemia, hyperparathyroidism, pregnancy, lactating women, and urinary tract stone were excluded from the study. Discontinuation criteria included unwillingness to continue study and intolerable side effects or allergy. All participants were examined physically, and their clinical history was noted. Age, weight, body mass index (BMI), and age were recorded.

### 2.3. Randomization and Outcomes

Enrolled participants were selected using block randomization and allocated to single or split dose groups by computer program. Primary outcome comprised the change of serum 25(OH)D concentrations at eight weeks after supplementation. The serum 25(OH)D level at the end of study was measured at 1 week after last dose of ergocalciferol by electrochemiluminescence binding assay, intended for use on Elecsys and Cobase 601 immuno-assay analyzers. Serum calcium, serum phosphate, and intact PTH levels were monitored before and after treatment. Safety outcomes included treatment-related adverse event, hypercalcemia, and hyperphosphatemia. All subjects were observed for adverse effects of excess vitamin D supplementation and hypercalcemic symptoms such as polydipsia, polyuria, dry mucosa membrane, vomiting, fatigue, anorexia, weakness, weight loss, and decreased appetite. The researcher verified consistent vitamin D supplementation by asking for the remaining tablets and followed up the side effects of vitamin D supplementation by using the adverse effects assessment form (Naranjo's algorithm).

### 2.4. Statistical Analyses

Numerical data were expressed as mean ± standard deviation (SD) for normally distributed data or median (interquartile range, IQR) for skewed data, and categorical data were expressed as a count with number (*N*) and percentage (%). Comparisons used the paired *t*-test for continuous variables within group and Student's *t*-test for continuous variables between groups. Categorical variables were compared using the chi-square test. Statistical inferences were made on the basis of a two-sided significance level of *P* < 0.05. All analyses were performed using SPSS, Version 13.0 for Windows (SPSS, Inc., Chicago, IL, USA).

## 3. Results

**Figure 2:**
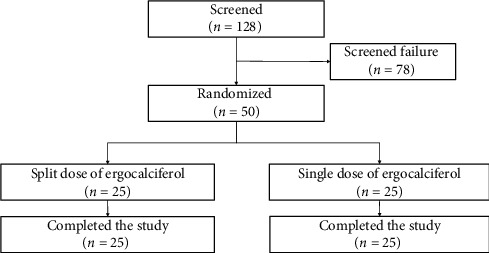
Study flow diagram.

One hundred and twenty-eight patients on CAPD were screened from January 2018 until February 2019. A total of 50 patients met the criteria for eligibility and were randomly assigned to receive single or split doses of ergocalciferol 20,000 IU capsules three times weekly (one capsule of ergocalciferol with two capsules of placebo on Monday and one capsule of ergocalciferol on Wednesday and Friday) (*N* = 25). The single dose group was given ergocalciferol 60,000 IU weekly (three capsules of ergocalciferol on Monday and one capsule of placebo on Wednesday and Friday) (*N* = 25) for eight weeks, as shown in Figure 2. All patients (100%) completed the eight-week therapy and were included in the analysis. Patients in both groups had a medication possession ratio of 100%, indicating good adherence.

Baseline clinical characteristics and laboratory results of patients were similar in both groups, as shown in Table 1. Mean age was 62.4 ± 15.3 years, and the proportion of men was 54%. Mean BMI was 23.3 ± 4.7 kg/m^2^. The main underlying disease was hypertension (100%). The mean duration to undergo peritoneal dialysis was 1.3 ± 1.8 years. Baseline serum 25(OH)D level, calcium, phosphate, and intact PTH levels were 22.8 ± 5.5 ng/mL, 9.1 ± 0.6 mg/dL, 4.5 ± 1.3 mg/dL, and 263.4 ± 217.1 pg/mL, respectively. Baseline serum 25(OH)D level in single and split dose ergocalciferol was 22.9 ± 5.3 and 22.7 ± 5.9 ng/mL (*P*=0.977), respectively.

## 4. Outcomes

**Figure 3:**
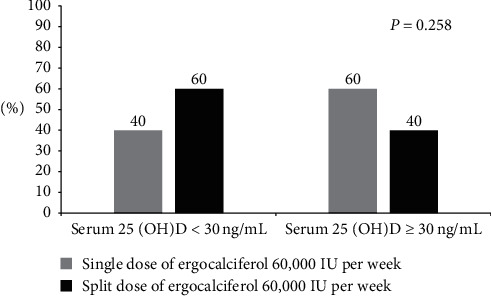
Serum 25-OH-D status after treatment with single and split dose ergocalciferol 60,000 IU weekly.

At eight weeks of treatment, mean serum 25(OH)D concentrations significantly increased from baseline 22.7 ± 5.9 to 29.5 ± 9.5 ng/mL (*P*=0.004) in the split dose group and 22.9 ± 5.3 to 31.2 ± 12.3 ng/mL (*p*=0.003) in the single dose group. The mean absolute increase ± SD of serum 25(OH)D at 8 weeks did not differ between the single and the split dose groups (8.4 ± 11.9 vs. 6.8 ± 10.5 ng/mL, respectively, *P*=0.561) (Table 2). At the end of the study, a similar proportion of patients in both groups reached the desirable serum concentration of 25(OH)D ≥ 30 ng/mL (60% in the single group vs. 40% in the split group, *P*=0.258) (Figure 3).

No statistically significant change was detected in serum phosphorus, calcium, and intact PTH levels between baseline and at eight weeks. Moreover, no participants experienced hypercalcemia or hyperphosphatemia and no serious adverse events occurred during the study.

## 5. Discussion

This randomized double-blind controlled trial investigated the efficacy of split and single doses of ergocalciferol 60,000 IU weekly to treat vitamin D deficiency and insufficiency among patients on CAPD. At eight weeks after treatment, both groups significant increased serum 25(OH)D compared with baseline, but there were no different outcomes between groups.

Vitamin D deficiency and insufficiency are frequent disorders in a CKD population [3], and serum 25(OH)D level is lower among patients on CAPD and hemodialysis compared with patients with CKD [14]. Low dietary vitamin D intake and minimal sun exposure commonly causes hypovitaminosis D among patients on long-term dialysis including CAPD. Oral ergocalciferol can be taken once daily but also at longer intervals because it has an average half-life of 24 hours and a duration of up to 6 months. The sustained effect of high-dose vitamin D may be attributed to its long half-life and converted slow release 25(OH)D form. Several studies have been undertaken to evaluate and determine the intake of vitamin D supplement needed to attain and maintain the optimal serum 25(OH)D level in the general population [15–18]. Few studies have evaluated the optimal dose and time intervals of oral vitamin D supplementation to improve 25(OH)D status in CKD populations [12, 19, 20]. Our study indicated that both regimens of oral vitamin D supplementation appeared to be effective, resulting in increased serum 25(OH)D levels. These results were similar to one study reporting that supplementation with vitamin D3 achieved equally well with daily, weekly, or monthly dosing frequencies in an elderly population [21], and one study also found that daily and monthly vitamin D3 supplementation exhibited equivalent adherence and improvements in vitamin D status in a CKD population [19].

Our study showed that ergocalciferol 60,000 IU weekly increased serum 25(OH)D levels without change of serum calcium, phosphate, and PTH at eight weeks. Similarly, several randomized controlled trials found that oral ergocalciferol or cholecalciferol increased serum 25(OH)D levels among advanced CKD patients and those on hemodialysis without significant alterations in plasma calcium, phosphate, or PTH [22–26]. However, a systemic review and meta-analysis indicated that vitamin D supplement was efficient for restoring 25(OH)D levels with a decrease in serum PTH level and low incidence of hypercalcemia and hyperphosphatemia among patients on CKD and dialysis [27]. The discrepancies may be due to differences in baseline serum 25(OH)D levels, vitamin D dosage, type of vitamin D supplement, duration of the study, patient population, and comorbid illness.

Our study encountered several limitations. The present study was limited by the short duration of follow-up without apparent treatment-related safety and benefits in preventing metabolic and bone outcomes among patients on CAPD at an academic medical center in Bangkok, Thailand. Our population may not be representative of the general CAPD population and may limit generalizability to other regions. Several studies have reported that cholecalciferol was more effective at raising serum 25(OH)D among healthy patients and those on CKD compared with ergocalciferol [28, 29]. We selected ergocalciferol because of being an easily available nutritional vitamin D supplement in Thailand, so our outcomes may not be generalizable to cholecalciferol.

## 6. Conclusion

Because of the long life of complex 25(OH)D and vitamin D binding protein, single and split doses of ergocalciferol 60,000 IU weekly efficiently restored 25(OH)D levels at eight weeks and 60% of the single dose group reached desirable concentrations of 25(OH)D, suggesting that single dose weekly might be required for adequate vitamin D repletion among patients on CAPD.

## Figures and Tables

**Table 1:** Baseline characteristics.

Characteristics	Total (*N* = 50)	Ergocalciferol split dose (*N* = 25)	Ergocalciferol single dose (*N* = 25)	*P* value
Male (*N*, %)	23 (46)	13 (52.0)	10 (40.0)	0.395
Age (years)	62.4 ± 15.3	61.1 ± 16.3	63.8 ± 14.6	0.541
Body mass index (kg/m^2^)	23.3 ± 4.7	22.8 ± 4.8	23.8 ± 4.7	0.453
Hypertension	50 (100)	25 (100)	25 (100)	0.999
Diabetes mellitus	11 (22)	2 (8.0)	9 (36.0)	0.017
Dyslipidemia	15 (30)	5 (20.0)	10 (40.0)	0.123
Time of initiate CAPD (years)	1.3 ± 1.8	1.2 ± 1.6	1.5 ± 2.1	0.646
History infected CAPD	7 (14)	2 (8.0)	5 (20.0)	0.417
25(OH)D ≤ 20 ng/mL (*N*, %)	18 (36.0)	9 (36.0)	9 (36.0)	0.999
25(OH)D 21–30 ng/mL (*N*, %)	32 (64.0)	16 (64.0)	16 (64.0)	0.999
Serum 25(OH)D (ng/mL)	22.8 ± 5.5	22.7 ± 5.9	22.9 ± 5.3	0.977
Serum calcium (mg/mL)	9.1 ± 0.6	9.2 ± 0.7	9.1 ± 0.53	0.457
Serum phosphate (mg/mL)	4.5 ± 1.3	4.7 ± 1.4	4.30 ± 1.17	0.266
Intact parathyroid hormone (pg/mL)	263.4 ± 217.1	266.7 ± 255.6	270.0 ± 188.1	0.522

Data in the table are shown with average ± standard deviation and percentages. CAPD, continuous ambulatory peritoneal dialysis; 25(OH)D, 25-hydroxyvitamin D.

**Table 2:** Outcomes between split and single dose of ergocalciferol 60,000 IU weekly supplement at 8 weeks.

Parameters	Split dose of ergocalciferol (*N* = 25)			Single dose of ergocalciferol (*N* = 25)			*P* value^B^
	Baseline	At 8 weeks	Mean change	Baseline	At 8 weeks	Mean change	
Serum 25(OH)D (ng/mL)	22.7 ± 5.9	29.5 ± 9.5^A^	6.8 ± 10.5	22.9 ± 5.3	31.2 ± 12.3^A^	8.4 ± 11.9	0.561
Serum calcium (mg/dL)	9.2 ± 0.7	9.5 ± 0.6	0.3 ± 0.7	9.1 ± 0.5	9.3 ± 0.6	0.3 ± 0.8	0.276
Serum phosphate (mg/dL)	4.7 ± 1.4	4.5 ± 1.1	−0.2 ± 1.5	4.3 ± 1.2	4.2 ± 1.1	−0.2 ± 1.2	0.912
Intact parathyroid hormone (pg/mL)	266.7 ± 255.6	220.0 ± 172.9	46.7 ± 188.7	270.0 ± 188.1	260.2 ± 175.7	−9.9 ± 114.3	0.206

Data in the table are shown with average ± standard deviation. 25(OH)D, 25-hydroxyvitamin D. ^A^*P* value < 0.05 vs. baseline. ^B^*P* value of mean change between the two groups.

## Data Availability

The excel data used to support the findings of this study are available from the corresponding author upon request.

## Abbreviations

BMI: Body mass index
CKD: Chronic kidney disease
CAPD: Continuous ambulatory peritoneal dialysis
IQR: Interquartile range
PTH: Parathyroid hormone
SD: Standard deviation
25(OH)D: 25-Hydroxyvitamin D.
